# Phytase activity in lichens

**DOI:** 10.1111/nph.13454

**Published:** 2015-05-12

**Authors:** Niall F. Higgins, Peter D. Crittenden

**Affiliations:** ^1^ School of Life SciencesUniversity of NottinghamNottinghamNG7 2RDUK

**Keywords:** enzymes, epiphytes, *Evernia prunastri*, inositol hexaphosphate, phosphomonoesterase, phytic acid

## Abstract

- Phytase activity was investigated in 13 lichen species using a novel assay method. The work tested the hypothesis that phytase is a component of the suite of surface‐bound lichen enzymes that hydrolyse simple organic forms of phosphorus (P) and nitrogen (N) deposited onto the thallus surface.
- Hydrolysis of inositol hexaphosphate (InsP_6_, the substrate for phytase) and appearance of lower‐order inositol phosphates (InsP_5_–InsP_1_), the hydrolysis products, were measured by ion chromatography. Phytase activity in *Evernia prunastri* was compared among locations with contrasting rates of N deposition.
- Phytase activity was readily measurable in epiphytic lichens (e.g. 11.3 μmol InsP_6_ hydrolysed g^−1^ h^−1^ in *Bryoria fuscescens*) but low in two terricolous species tested (*Cladonia portentosa* and *Peltigera membranacea*). Phytase and phosphomonoesterase activities were positively correlated amongst species. In *E. prunastri* both enzyme activities were promoted by N enrichment and phytase activity was readily released into thallus washings. InsP_6_ was not detected in tree canopy throughfall but was present in pollen leachate.
- Capacity to hydrolyse InsP_6_ appears widespread amongst lichens potentially promoting P capture from atmospheric deposits and plant leachates, and P cycling in forest canopies. The enzyme assay used here might find wider application in studies on plant root–fungal–soil systems.

## Introduction

Lichens typically grow in habitats with low availabilities of nitrogen (N) and phosphorus (P) (Crittenden, 1989; Crittenden *et al*., 1994). There is evidence to suggest that supply of these key nutrients is growth limiting for the more productive lichens such as mat‐forming species of *Cladonia* and large foliose cyanolichens (Crittenden *et al*., 1994; Benner & Vitousek, 2007; Benner *et al*., 2007; Kytöviita & Crittenden, 2007; McCune & Caldwell, 2009). The majority of lichens acquire these nutrients from atmospheric deposits by efficiently scavenging inorganic N and P (Pi) from solutes in precipitation intercepted either directly or after being modified by plant canopies and then delivered to thalli as canopy throughfall and stem flow (Crittenden, 1983, 1989, 1998). Nitrogen compounds are routinely monitored in both precipitation and air, and there is an abundance of data on rates of N deposition with which lichen N concentration ([N]) is frequently positively correlated (Bruteig, 1993; Hyvärinen & Crittenden, 1998b; Remke *et al*., 2009; Hogan *et al*., 2010a; Boltersdorf *et al*., 2014). By contrast, P is not routinely measured in atmospheric deposits, there is a dearth of information on P deposition rates and how lichens respond to variation in P income is largely unknown. Detailed P‐focused investigations suggest that Pi concentration in rainfall is in the range 0.03–0.33 μmol l^−1^ (Grimshaw & Dolske, 2002; Neal *et al*., 2003; Yoshioka *et al*., 2009; He *et al*., 2011); Pi at these low concentrations is taken up rapidly by lichens via high affinity phosphate transport systems (Farrar, 1976; Hogan, 2009).

In addition to a capacity for rapid uptake of inorganic ions and small nitrogenous organic molecules such as amino acids (Dahlman *et al*., 2004), lichens also have surface phosphatase activities that promote the release of covalently bonded P in organophosphates (Po) (Lane & Puckett, 1979; Hogan *et al*., 2010a; Crittenden *et al*., 2015). Artificial chromogenic Po substrates hydrolysed by lichens include the monoester *para*‐nitrophenyl phosphate (*p*NPP), and the diesters bis‐ (*para)* nitrophenol phosphate (*bis‐p*NPP) and *para‐*nitrophenyl phenylphosphonate (*p*NPPP, a DNA analogue) (Hogan, 2009); the hydrolysis of these compounds can be measured colorimetrically because it results in the coloured product, *para*‐nitrophenol (*p*NP). Capacity for phosphatase activity in lichens varies among both species and habitats probably reflecting differences in the availability of, and demand for, P (Lane & Puckett, 1979; Hogan, 2009; Lewis, 2012). N enrichment, due to anthropogenic pollution, alters thallus N : P ratio driving an upregulation of phosphomonoesterase (PME) activity (Hogan, 2009; Hogan *et al*., 2010a,b). However, despite the apparent diversity of phosphatase activities as indicated by analogous substrates, little is known about the forms and quantities of Po available to lichens in their natural habitats (cf. Tibbett, 2002; Turner *et al*., 2002).

Most natural waters to which lichens are exposed contain traces of organic P. Yoshioka *et al*. (2009) and He *et al*. (2011) found the ratio of total P : soluble reactive phosphate‐P was between 3 and 5 in both rainfall and atmospheric aerosol, raising the possibility of a significant organic P component in atmospheric deposits. Annual deposition of this putative Po fraction was in the range 11–50 and 38–160 mg m^−2^ yr^−1^ for wet and dry inputs, respectively (He *et al*., 2011). However, specific compounds were not identified. Rainfall that passes through plant canopies as throughfall and stemflow can be enriched in organic carbon including organophosphates (Zimmermann *et al*., 2007). Again specific compounds have not been identified but these probably include a range of organophosphate metabolites from plant leaves, epiphytes and phylloplane microorganisms. Also, lichens that grow on soil, or on surfaces exposed to seepage water draining from soil, will be exposed to traces of soil organic P (Martinsen *et al*., 2013); most lichens can potentially experience deposition of animal faeces or solutes derived from them (Tomassen *et al*., 2005).

Phytic acid (inositol hexa*kis*phosphate; InsP_6_) is a major source of Po in the environment (Turner *et al*., 2002; Turner, 2006). InsP_6_ is a *myo*‐inositol ring esterified with six symmetrically distributed phosphate groups (Shears & Turner, 2007). Lower‐order inositol phosphates (InsP_5_–InsP_1_), distinguished by the number of substituted phosphate groups on the inositol ring, also occur widely in nature. InsP_6_ is a common constituent of eukaryotic cells where it is involved in signal transduction and ATP regeneration, amongst other processes (Raboy, 2003). It is also accumulated by plants (Frank, 2013), and to a lesser extent by microorganisms (Cosgrove & Irving, 1980; Turner *et al*., 2002), as a P storage compound. InsP_6_ accounts for up to 90% of total organic P in seeds (Frank, 2013) but also occurs in leaves, roots, flowers and fruits, constituting up to 6% of organic P in leaves of herbaceous plants (Alkarawi & Zotz, 2014a,b), up to 0.22% of total P in coniferous leaves (Frank, 2013) and up to 70% of organic P in pollen (Jackson *et al*., 1982). Data on InsP_6_ concentrations in microorganisms are extremely scant, reported values include 76 nmol g^−1^ in *Neurospora crassa* (Lakin‐Thomas, 1993) and 700 μM in the cytoplasm of *Dictyostelium discoideum* (Martin *et al*., 1987). Concentrations in lichens and bryophytes have not been documented, but Winkler & Zotz (2009) present evidence of InsP_6_ accumulation in the epiphytic bromeliad *Aechmea fasciata*. InsP_6_ becomes strongly complexed in soils during decomposition forming up to 60% of total soil Po reserves (Cosgrove & Irving, 1980; Turner *et al*., 2002, 2007; Turner, 2006).

Although InsP_6_ is likely to prove an important component of the terrestrial P cycle, there remains a dearth of information regarding the rates of release of Pi during its degradation (Turner *et al*., 2002). Phytases (*myo*‐inositol hexa*kis*phosphate phosphohydrolases) are probably the principal agents involved in hydrolysis of the ester linkages. This class of enzyme initiates a stepwise dephosphorylation of InsP_6_, releasing Pi and a series of partially dephosphorylated *myo*‐inositols. Phytases have been most frequently reported in fungi and bacteria (Mukhametzyanova *et al*., 2012) but are also produced by plants (Hayes *et al*., 1999), most notably in germinating seeds (Gibson & Ullah, 1988; Hegeman & Grabau, 2001). Studies of phytase activity have been impeded by the lack of a sensitive assay. Inositol phosphates do not absorb visible or ultraviolet light and therefore cannot be detected spectrophotometrically. A new chromophoric substrate analogue of InsP_6_, 5‐O‐[6‐(benzoylamino)hexyl]‐d‐*myo*‐inositol‐1,2,3,4,6‐ penta*kis*phosphate (T‐IP5), is under development; this permits the measurement of phosphate ester bond cleavage by phytase (Berry & Berry, 2005; Berry *et al*., 2007) but the authors consider that further refinement of the method is required before it can be used to assay biologically active samples. To date, measurement of phytase activity has largely relied on determining the rate of Pi release during InsP_6_ hydrolysis (Antibus *et al*., 1992; Yadav & Tarafdar, 2003). However, this method suffers from the drawback that living material, such as lichens or mycorrhizal roots, frequently has a high Pi uptake rate (Farrar, 1976; Hyvärinen & Crittenden, 1998a; Smith & Read, 2008; Hogan, 2009) which potentially will lead to underestimation of Pi release promoted by phytase.

The aim of the present study was to test for the presence of phytase activity in lichens employing a method that did not rely on the measurement of Pi release from samples. Accordingly, we use a high performance ion chromatographic method to measure the rates of consumption of InsP_6_ and the production of lower‐order inositol phosphates. We develop a phytase assay procedure suitable for lichens using the common epiphyte *Evernia prunastri* and then compare rates of activity among 13 lichens including 11 epiphytic and two terricolous species. We also test the hypothesis that phytase activity responds positively to N enrichment as has been demonstrated for PME activity in lichens (Hogan *et al*., 2010a; Crittenden *et al*., 2015). The results are discussed in relation to possible sources of InsP_6_ for lichens in their natural habitats.

## Materials and Methods

### Lichen collection and pretreatment

*Evernia prunastri* was collected from the Derwent Valley, Derbyshire, UK, and from other sites subject to different rates of wet inorganic N (NO_3_^−^ + NH_4_ ^+^) deposition (Table 1). Modelled mean N deposition values pertaining to the collection sites for the period 2009–2011 were provided by R.I. Smith (Centre for Ecology and Hydrology, Edinburgh, UK) and were abstracted from 5 × 5 km gridded datasets. A further 12 species were collected for an interspecies comparative study (Table 1).

**Table 1:** Lichen species studied, details of collection sites in the UK including values of modelled annual mean wet deposited inorganic nitrogen (N) (mean for 2009–2011), and putative optimum pH values used in enzyme assays

Species	Collection site	National Grid Reference	N deposition (kg ha^−1^ yr^−1^)	pH optimum and source
*Evernia prunastri* (L.) Ach.	Lough Erne, Co. Fermanagh	NV 193243	5	2.5, determined in present study
	Elan Valley, Powys	SN 975574	9.4	
	Beddgelert, Gwynedd	SH 575509	13	
	Greystoke Forest, Cumberland	NY 402335	17	
	Derwent Valley, Derbyshire	SK 147931	25.6	
	Derwent Fells, Cumberland	NY 208192	26.5	
	Tarn at Leaves, Cumberland	NY 259124	29.7	
	Naddle Forest, Cumberland	NY 493149	30	
	Wythburn Fells, Cumberland	NY 327135	37.1	
*Lobaria pulmonaria* (L.) Hoffm.	Lairg, Sutherland	NC 476021	4.2	4.8, surface pH, present study
*Ramalina fraxinea* (L.) Ach.	Kildonan, Sutherland	NC 976186	4.3	3.2, surface pH, present study
*Bryoria fuscescens* (Gyeln.) *Brodo & D. Hawksw*.	The Halsary, Caithness	ND 195506	4.7	2.5, surface pH, present study
*Pseudevernia furfuracea* (L.) Zopf	The Halsary, Caithness	ND 195506	4.7	2.5, surface pH, present study
*Ramalina calicaris* (L.) Fr.	The Halsary, Caithness	ND 195506	4.7	3.2, surface pH, present study
*Peltigera membranacea* (Ach.) Nyl	Beddgelert, Gwynedd	SH 575509	13	4.3, determined in present study
*Cladonia portentosa* (Dufour) Coem.	Migneit, Gwynedd	SH 749433	12.1	2.5, Hogan *et al*. (2010a)
*Hypogymnia physodes* (L.) Nyl.	Derwent Valley, Derbyshire	SK 147931	25.6	3.2, Lewis (2012)
*Parmelia saxatilis* (L.) Ach.	Derwent Valley, Derbyshire	SK 147931	25.6	3.4, surface pH, present study
*Platismatia glauca* (L.) W.L. Culb & C.F. Culb	Derwent Valley, Derbyshire	SK 147931	25.6	3.0, Lewis (2012)
*R. farinacea* (L.) Ach.	Derwent Valley, Derbyshire	SK 147931	25.6	4.8, surface pH, present study
*Usnea subfloridana* Stirt.	Derwent Valley, Derbyshire	SK 147931	25.6	3.2, surface pH, present study

At each collection site, 10 replicate lichen samples were collected from locations > 10 m apart. Samples were returned to the laboratory in polythene bags, air dried on a laboratory bench for 24 h at 18°C, re‐sealed in polythene bags and stored at −20°C until required. Before assays, lichens were gradually rehydrated overnight in water‐saturated air (over water in a desiccator) at 10°C, then fully re‐saturated by spraying with deionized water, cleaned of extraneous debris and then blotted to remove surface moisture. In order to reduce variability among replicates, assays were confined to either the terminal 10‐mm branch tips of fruticose lichens or the marginal 10 mm of foliose thalli; these were cut off using a scalpel and used immediately in assays (see enzyme assays below). Powder‐free latex gloves were used at all times when handling lichens in the field and laboratory.

### Thallus surface pH

Thallus surface pH was measured following Hogan *et al*. (2010a). In brief, a flat tip pH electrode (Gelplas double junction flat tip electrode; VWR International Ltd, Lutterworth, UK) was held in contact with the apical or marginal 15‐mm region of the lichen thallus, saturated with excess 0.025 M KCl, noting the pH value after 1 min.

### Determination of phytase activity

Assays were initiated by adding *c*. 40 mg (unless otherwise stated) wet mass of lichen thallus to 3 ml of InsP_6_ (dodecasodium salt hydrate; Sigma‐Aldrich) solution made up in citric acid‐trisodium citrate buffer containing major ions representative of UK precipitation (20 μmol l^−1^ MgSO_4_.7H_2_O, 8 μmol l^−1^ CaCl_2._2H_2_O, 150 μmol l^−1^ NaCl, 15 μmol l^−1^ NH_4_NO_3_, 5 μmol l^−1^ KNO_3_; Hayman *et al*., 2007). The citric acid‐trisodium citrate buffer was prepared following Dawson *et al*. (1986) and then diluted 10‐fold. Samples were incubated in a shaking water bath in the dark at temperatures and for time periods ranging from 5 to 30°C and 0.25 to 24 h, respectively. Reactions were terminated by transferring 2.5 ml of the assay medium to 250 μl of 0.5 M HCl in an autosampler tube. Thalli were then blotted to remove surface solution and dried at 80°C for 24 h before weighing.

Quantities of InsP_6_ and its hydrolysis products in the acidified subsamples were determined by high performance ion chromatography (HPIC) following Chen & Li (2003) using a Dionex ICS5000 ion chromatograph (Dionex (UK) Ltd, Camberley, Surrey, UK). In brief, inositol phosphates were separated on a CarboPac^™^ PA‐100 analytical column (250 × 4 mm) at 30°C using 0.5 M HCl as mobile phase in a linear gradient elution program followed by post‐column reaction with a solution of 1 g l^−1^ Fe(NO_3_)_3_ in 0.33 M HClO_4_ using a Dionex knitted coil (750 μl). InsP_6,_ Ins(1,2,4,5,6)P_5_ and Ins(1,5,6)P_3_ peaks were identified from retention times of standards and InsP_4,_ InsP_2_ and InsP_1_ peaks were identified from retention times given by Chen & Li (2003), although specific isomers of InsP_2_ and InsP_1_ compounds were difficult to resolve. The system was calibrated for InsP_6_, the response being linear up to 0.1 μmol on column. Chromeleon 7.2 software (Dionex) was used for instrument control and data acquisition and processing. Run time required for InsP_6_, the slowest eluting analyte, was 50.1 min with an equilibration time of 10 min needed between each run. Rates of activity were expressed as μmol InsP_6_ hydrolysed g^−1^ dry mass h^−1^. ‘No‐lichen’ and ‘no‐substrate’ control samples were included in each batch of analyses.

Preliminary experiments examined phytase response to pH (2.5–6.7), and to substrate concentration (1–10 mM); rates from the latter experiment were fitted to the Michaelis–Menten equation using OriginPro 8. This range of substrate concentrations was selected to help characterize the enzyme system and has no ecological relevance (cf. Whitton *et al*., 2005) because in nature InsP_6_ is likely to be available to lichens in trace quantities only. The assay medium pH value selected for each species was either the putative optimum value determined in this study, the optimum value for PME activity determined by Lewis (2012) or the surface pH value (Table 1). The potential inhibitory effect on phytase activity of *p*NPP, the substrate for PME assays (see Determination of phosphomonoesterase activity below), was investigated in *E. prunastri* by including both *p*NPP and InsP_6_ in phytase assays in equimolar concentrations.

In order to determine whether enzyme activity could be removed from the thallus by washing, *c*. 150 mg dry mass of *E. prunastri* was shaken in 10 ml of assay medium at the optimum pH, and 15°C for 5 h. Phytase assays were then run on the following samples: (1) thalli removed from the wash and blotted dry with tissue to remove old assay medium (= washed thalli); (2) 2.9 ml of assay medium without thalli (= washings); (3) 2.9 ml of filtered (to pass 0.22 μm) assay medium without thalli (= filtered washings); and (4) fresh thalli not pre‐treated by washing (= unwashed thalli). Washings were filtered to exclude lichen‐associated bacteria and yeasts that might be a source of phytase (Mukhametzyanova *et al*., 2012). Assays on washings were initiated by adding 0.1 ml InsP_6_ solution to 2.9 ml to yield a final substrate concentration of 1 mM and incubated for 1 h. Phytase activity in washings was expressed per unit mass of thallus from which the washings were derived.

### Determination of phosphomonoesterase activity

PME activity was determined using Bessey *et al*.'s (1946) *p*NPP colorimetric assay as described by Hogan *et al*. (2010a). Lichen samples were added to 2.9 ml assay medium of similar composition to that used above for phytase measurements. Assays were initiated by adding 0.1 ml *p*NPP, to yield a final concentration of either 0.5 or 10 mM and samples were incubated for 20 min as for phytase assays, after which the reaction was terminated by transferring 2.5 ml assay medium into 0.25 ml terminator solution (1.1 M NaOH, 27.5 mM EDTA, 0.55 M K_2_HPO_4_) and the absorbance measured at 405 nm using a NanoDrop ND‐1000 spectrophotometer (Thermo Fisher Scientific, Waltham, MA, USA). Thalli were then dried and weighed as above. Effective enzyme activity was expressed as μmol *p*NPP hydrolysed g^−1^ dry mass h^−1^ using *p*NP to calibrate the assay. ‘No‐lichen’ and ‘no‐substrate’ control runs were included in each batch of analyses. Assay medium pH was adjusted within the range 2.5–6.7 depending on species being tested; in each case the value selected was either the optimum value for the species determined by Lewis (2012), the optimum value for phytase activity or, if unknown, the thallus surface pH value (Table 1). Note that a 10 mM substrate concentration is considered saturating for PME activity in lichens (Hogan, 2009; Crittenden *et al*., 2015).

### Assessment of canopy throughfall and pollen as sources on InsP_6_

Canopy throughfall was sampled in triplicate in two stands of sycamore (*Acer pseudoplatanus*): during September in Nottingham City (Grid Reference SK 518375, 52°55′58′′N, 1°13′48′′W); and in October in the Derwent Valley, Derbyshire (Table 1) where trees support large populations of *E. prunastri*. Polyethylene funnels (diameter 22 cm) connected to 1‐l bottles were positioned beneath trees with the funnel tops at 1 m above the ground. Funnels were fitted with stainless steel spikes on their circumferences to prevent bird perching and resultant contamination (Asman *et al*., 1982); they were deployed shortly before the onset of forecast rainfall events and left in the field for up to 24 h. Throughfall samples were passed in sequence through a cellulose filter paper and a nitrocellulose membrane filter (0.22 μm, Millipore), freeze‐dried, and the residue stored at −20°C until required. Samples were then re‐solubilized in 1 ml water and analysed by HPIC for the presence of InsP_6_.

Pollen (25 mg) was collected from oriental pink lilies (*Lilium candidum*), and shaken in 7 ml of assay medium at the optimum pH for 1 h. Filtrate (0.22 μm, Millipore) was analysed as above. To determine whether enzyme activity in *E. prunastri* could hydrolyse InsP_6_ present in lily pollen, lichen thalli were incubated in assay medium containing pollen for 5 h and then analysed as described above.

### Data analysis

SPSS (SPSS (UK) Ltd, Woking, UK) was used to perform standard statistical analyses. All data were checked for normality of distribution (Kolmogorov–Smirnov test) and homogeneity of variances and where test assumptions were not met, either log‐ or square root‐transformed. Relationships between rates of enzyme activity and environmental variables were subjected to either one‐way ANOVA, ANCOVA, correlation or regression analysis.

## Results

All raw data are available in Supporting Information Table S1.

### Studies on *Evernia prunastri*

**Figure 1:**
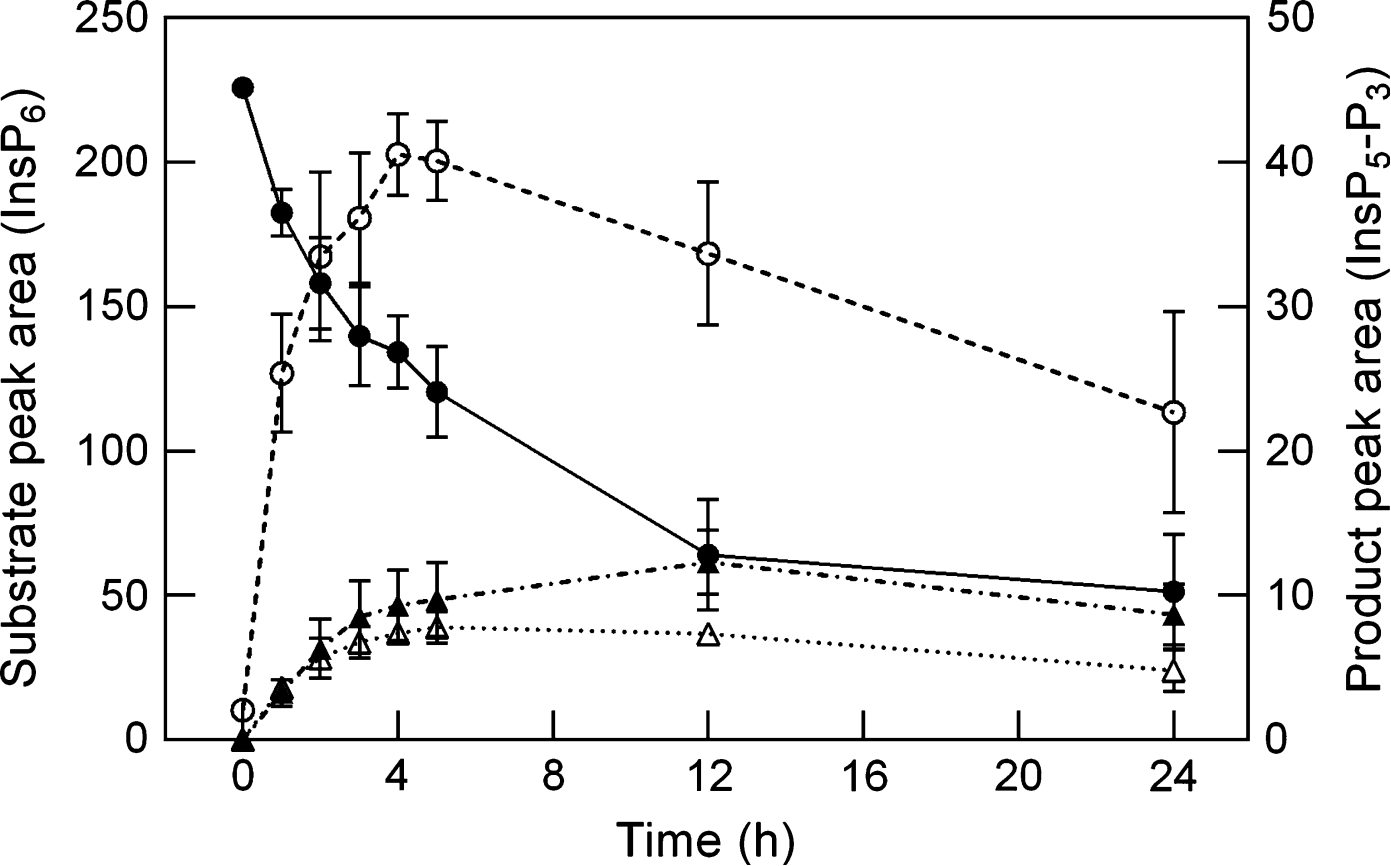
Progress of InsP_6_ hydrolysis and appearance of InsP_6_ hydrolysis products in the apical 10 mm segments of *Evernia prunastri* as measured by HPIC: InsP_6_, closed circles (left‐hand axis); InsP_5_, open circles; InsP_4_, open triangles; InsP_3_, closed triangle (right‐hand axis). Samples were incubated for increasing time periods up to 24 h in 1 mM InsP_6_ at pH 2.5 and 15°C in the dark. Plotted values are means (*n *=* *6) ± 1 SEM.

Hydrolysis of InsP_6_ was readily detectable in *Evernia prunastri*. As incubation time increased from 0.5 to 24 h, InsP_6_ decreased in abundance while a peak consistent with InsP_5_, the first hydrolysis product, appeared and rapidly increased. Peaks consistent with InsP_4_–InsP_1_ were also produced and similarly increased over a 24 h incubation period. Hence, a reduction in InsP_6_ abundance could be used as a measure of phytase activity (Fig. 1). The detection limit (= 5× standard deviation of repeat ‘blank’ samples; Wilson, 1974) for depletion of InsP_6_ in samples containing 1 μmol ml^−1^ InsP_6_ was 0.0018 μmol ml^−1^, roughly equating to a rate of activity in *E. prunastri* of 0.86 μmol InsP_6_ hydrolysed g^−1^ h^−1^. ‘No‐substrate’ controls did not produce substances that could be detected by HPIC.

**Figure 2:**
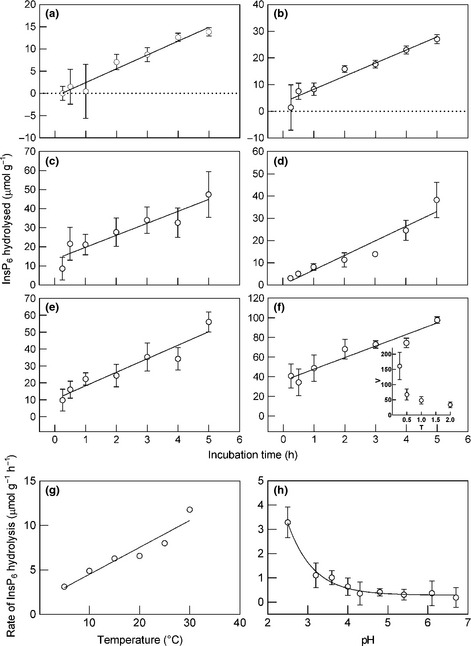
Effect of temperature and pH on InsP_6_ hydrolysis in the apical 10 mm of *Evernia prunastri*. Samples were incubated in 1 mM InsP_6_ in the dark; plotted values are means ± 1 SEM. (a–f) Progress of hydrolysis in incubations of different durations and at different temperatures (5–30°C), regression lines are indicated (assay pH = 2.5, *n *=* *6); insert in (f) shows selected data plotted as mean apparent rate of InsP_6_ hydrolysis (*V*, μmols g^−1^ h^−1^) determined in assays of increasing duration (*T*, h). (g) Effect of temperature on rates of InsP_6_ hydrolysis (*r* ^2^ = 0.915; *P *<* *0.01); plotted values are the gradients of regression lines in (a–f) and SE bars are less than the diameter of the plotted points. (h) Effect of pH on phytase activity at 15°C (*n *=* *10); rate of activity was determined from the difference between the quantities of InsP_6_ hydrolysed in 0.5‐ and 5‐h incubation periods; data are fitted to a two‐parameter exponential decay (*r* ^2^ = 0.977; *P *<* *0.01).

Although InsP_6_ was progressively hydrolysed with time, rates during the first 0.25 h were often atypically high. This was clearly evident when the progress of InsP_6_ hydrolysis in incubations of increasing duration was compared at different temperatures (Fig. 2a–f). However, the rates of hydrolysis determined from the gradients of the regression lines for each temperature were highly correlated with temperature (Fig. 2g) suggesting that initial high rates often observed in short assays might result from nonenzymic loss of substrate (e.g. Fig. 2f). Therefore, to avoid overestimation of enzyme activity, rate of phytase activity in all subsequent assays was determined from the difference in the quantity of InsP_6_ hydrolysed between 0.5 h and 5 h incubations.

Phytase activity was strongly dependent on both pH (Fig. 2h) and substrate concentration (Fig. S1). Maximum rates were recorded at pH 2.5 and were 18 times higher than those at pH 6.7; this putative optimum is broadly consistent with the measured thallus surface pH of 3.4 ± 0.1 (*n *=* *6). Phytase response to substrate concentration yielded apparent *K*_m_ and *V*_max_ values of 3.01 ± 0.5 mM and 13.08 ± 2.2 μmol InsP_6_ hydrolysed g^−1^ dry mass h^−1^, respectively. A non‐enzyme‐saturating substrate concentration of 1 mM was used in all subsequent routine assays to reduce costs and to obviate the need to routinely dilute samples before HPIC analysis.

**Figure 3:**
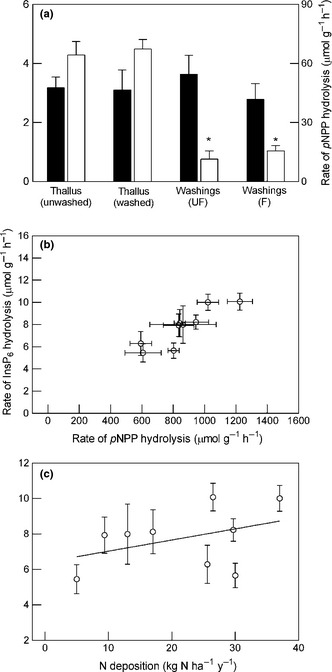
Variation in rates of InsP_6_ and *para*‐nitrophenyl phosphate (*p*NPP) hydrolysis in *Evernia prunsatri* among different washing treatments and different collection sites. (a) Rates of InsP_6_ hydrolysis (closed bars) and *p*NPP hydrolysis (open bars) in thalli (+ assay medium) and in thallus washings alone; samples were incubated in assay medium for 5 h and activity in the supernatant (= washings, unfiltered (UF) or filtered (F)) compared with that in thalli (either with or without a 5‐h washing pretreatment) (*, significantly different from thallus incubations; one‐way ANOVA with post‐hoc analysis). (b, c) Relationships between phytase activity and (b) phosphomonoesterase (PME) activity (*r* = 0.88, *P *<* *0.01), and (c) nitrogen (N) deposition (*r* ^2^ = 0.16, *P *>* *0.05); data points represent samples collected at sites subject to different rates of wet N deposition. In all cases assays were performed on the apical 10 mm of thalli in either 1 mM InsP_6_ or 10 mM *p*NPP and at pH 2.5 and 15°C in the dark. Rate of phytase activity in thalli was determined from the difference between the quantities of InsP_6_ hydrolysed in 0.5‐ and 5‐h incubation periods. Plotted values are means ± 1 SEM (*n *=* *6 (a) or 10 (b, c)).

Lichen washings contained appreciable phytase activity which was reduced little by filtering (Fig. 3a). Surprisingly, there were no significant differences between phytase activities in the filtered washings alone and in either fresh (i.e. not pre‐washed) lichen or lichen prewashed for 5 h. Lichen washings also contained PME activity, but rates of *p*NPP hydrolysis recorded in filtered and unfiltered washings were only 17–23% of those in assays containing thalli.

Phytase and PME activities in *E. prunastri* strongly co‐varied among sites with contrasting N deposition (Fig. 3b). However, neither enzyme activity was significantly related to wet inorganic N deposition (phytase activity, Fig. 3c; PME activity, data not shown, *r* ^2^ = 0.22, *P *=* *0.19) or to its components (deposition of NO_3_^−^ and NH_4_^+^ singly, and their concentrations in rainfall, data not shown). When samples were exposed to equimolar concentrations (0.5 mM) of InsP_6_ and *p*NPP, the rate of phytase activity was reduced by 48% (*P *=* *0.02, as revealed by ANOVA) compared to InsP_6_‐only treatments, whereas the rate of PME activ‐ity was reduced by 18% (*P *=* *0.38) compared to *p*NPP‐only treatments.

Collections of canopy throughfall from beneath sycamore trees were made during three rainfall events, each in triplicate. Throughfall volumes collected per funnel were in the range 220–490 ml. All nine collections were analysed by HPIC but this did not detect compounds that corresponded with either InsP_6_, InsP_5_ or InsP_4_, which have retention times > 18 min. Several unknown compounds with retention times < 18 min were detected; these were particularly abundant in late October when tree leaves were at an advanced stage of senescence/abscission.

**Figure 4:**
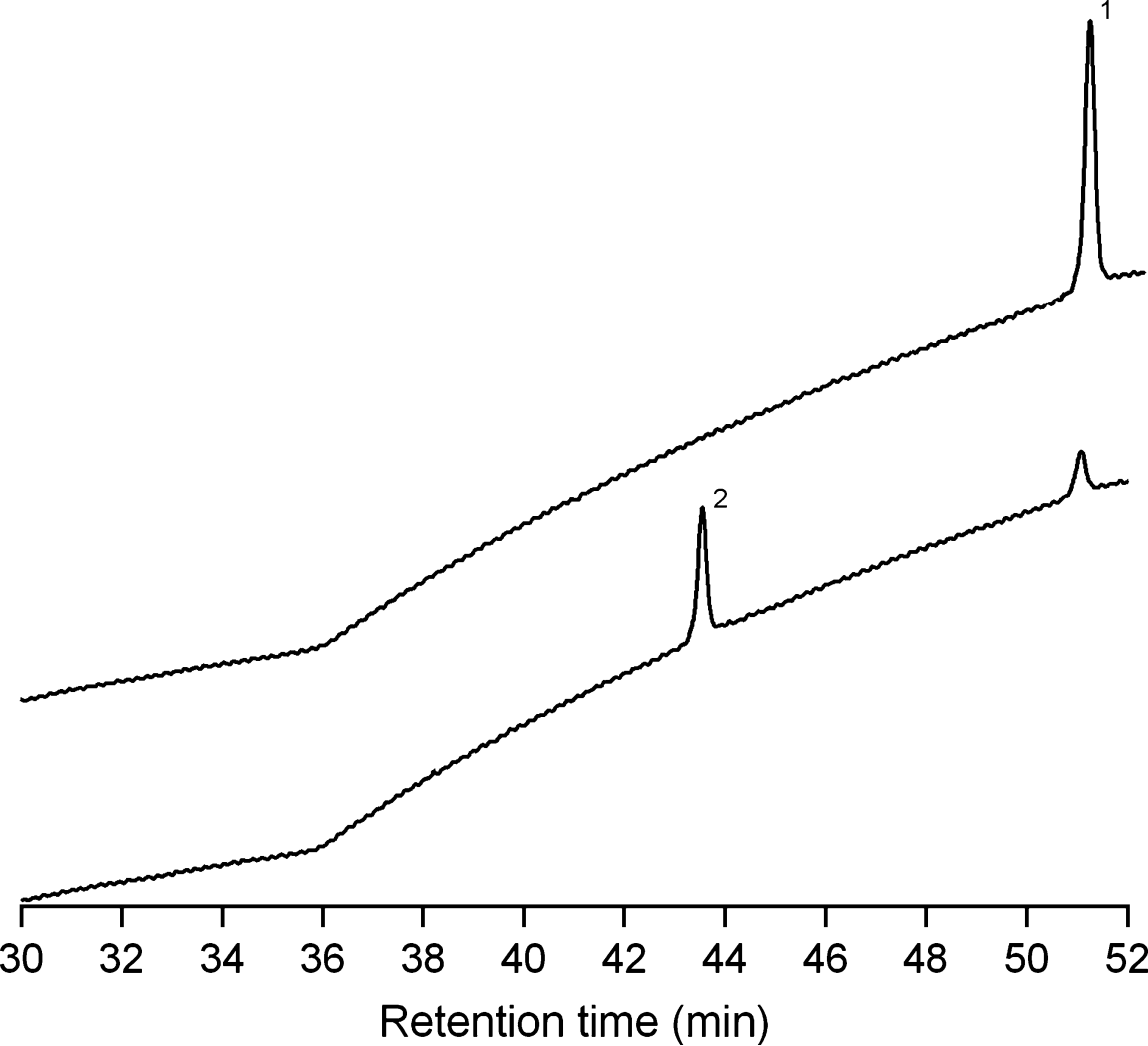
High performance ion chromatograms of lily pollen leachate. Upper chromatogram: leachate resulting from 25 mg of pollen shaken in 7 ml of assay medium at pH 2.5 for 1 h showing the presence of InsP_6_ (1). Lower chromatogram: leachate as above after 5 h incubation with apical 10 mm segments of *Evernia prunastri* showing the presence of InsP_5_ (2) and depleted InsP_6_.

InsP_6_ was readily detected in lily pollen leachate. Approximately 25 mg of pollen produced 57–74 μmols InsP_6_ in solution. When *E. prunastri* was incubated in filtered pollen leachate, InsP_6_ decreased in abundance concomitant with production of InsP_5_, the first hydrolysis product (Fig. 4).

### Interspecies comparisons of phytase and PME activities

**Figure 5:**
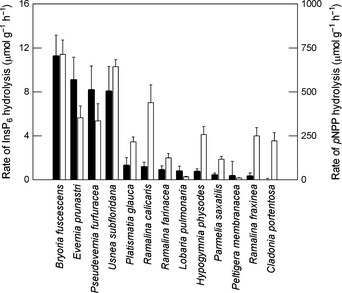
Rates of phytase (closed bars) and phosphomonoesterase (PME; open bars) activities in 13 lichen species. Assays were performed using either 1 mM InsP_6_ or 10 mM *para*‐nitrophenyl phosphate (*p*NPP), at 15°C in the dark and at the pH optimum pertaining to the species (Table 1). Rate of phytase activity was determined from the difference between the quantities of InsP_6_ hydrolysed in 0.5 and 5 h‐incubation periods. Plotted values are means (*n *=* *10) + 1 SEM.

Phytase activity was detected in the majority of lichen species tested with the highest rates in the epiphytic species *Bryoria fuscescens*,* E. prunastri, Usnea subfloridana* and *Pseudevernia furfuracea* (Fig. 5). In all cases where InsP_6_ depletion was detected, it was associated with production of InsP_5_. Lower phytase activity was measured in most other lichens with no activity being recorded in the terricolous mat‐forming lichen *Cladonia portentosa*. Phytase activity was one to two orders of magnitude lower than that of PME, but activities of the two enzymes among the 13 species tested were significantly correlated (*r *=* *0.77; *P *<* *0.01). For both enzyme activities the effect of species was significant at *P *<* *0.001 (ANCOVA).

## Discussion

We show for the first time that phytase activity is readily detectable in lichens. Rates of activity among the 13 species tested ranged from (0–) 0.3 to 11.3 μmol InsP_6_ hydrolysed g^−1^ h^−1^. These values are broadly comparable with those documented for mycorrhizal roots and mycorrhizal fungi in axenic culture which are generally in the range 1–20 μmol P released g^−1^ h^−1^ (Bartlett & Lewis, 1973; Straker & Mitchell, 1986; McElhinney & Mitchell, 1993; Antibus *et al*., 1997; Colpaert *et al*., 1997), although rates > 100 μmol P released g^−1^ h^−1^ have occasionally been reported (Antibus *et al*., 1992; Ahmad‐Ramli *et al*., 2013). The highest activities in lichens were recorded in epiphytic species, whereas those in the two terricolous lichens tested (*C. portentosa* and *Peltigera membranacea*) were the lowest. This might seem surprising for two reasons. First, there are large reserves of InsP_6_ in many soils (Turner *et al*., 2002, 2007, 2014), including podsols (Vincent *et al*., 2012), and thus a capacity to hydrolyse InsP_6_ could be advantageous in lichens growing in contact with soil. However, it has been argued that mat‐forming species of *Cladonia* (= subgenus *Cladina*) are largely insulated from soil chemistry by a basal layer of dead thallus or necromass (Crittenden, 1991) and Ellis *et al*. (2003, 2004) provided evidence to suggest that these terricolous species derive little N from soil and are largely dependent on atmospheric deposits for N supply. *Cladina* spp. typically occur in open habitats where rainfall is probably modified little by plant canopies. By contrast, stemflow and throughfall experienced by epiphytic lichens on trees are frequently enriched with organic compounds leached from the canopy above, especially during autumnal leaf senescence in deciduous forests (Le Mellec *et al*., 2010). Organic C in throughfall includes Po (Qualls & Haines, 1991; Qualls *et al*., 1991; Zimmermann *et al*., 2007) of which Qualls *et al*. (1991) speculate that inositol phosphates might be components. In the present work, InsP_6_ was not detected in canopy throughfall beneath sycamore trees supporting large populations of *E. prunastri*. Other compounds detected in throughfall could have included organophosphates and hence potential substrates for lichen PME, because the analytical system detects a range of Po compounds including glucose‐6‐phosphate, ATP, ADP and *p*NPP. Nonetheless, this analysis does not provide unambiguous evidence of Po in canopy throughfall because it also detects other anions such as sulphates and oxalate (Chen & Li, 2003). Lysing cells of microorganisms and pollen on the thallus surface are additional potential sources of InsP_6_. Pollen, in particular, contains high concentrations of InsP_6_ (2–3% of pollen mass) which may comprise up to 75% of total pollen Po (Jackson *et al*., 1982). Wind‐pollinated trees that dominate northern temperate and boreal regions produce significant quantities of pollen annually; for example, Saito and co‐workers have estimated pollen production on a dry mass basis in more than 10 forest types in Japan with values ranging from 55 to 96 kg ha^−1^ yr^−1^ in *Pinus densiflora*/*P. rigida* forests (Saito & Takeoka, 1985) to 70–356 kg ha^−1^ yr^−1^ in stands of *Quercus serrata* (Saito *et al*., 1991). Lee & Booth (2003) noted that pollen *deposition* in *P. densiflora* forests was only 30–50% of the estimated pollen *production* and proposed that this discrepancy might arise from pollen interception by trunks and leaves. Several authors have speculated that pollen deposition results in ecologically significant inputs of N and P to terrestrial and lake systems, with estimates of P inputs to pine forests in the range 1–7 mg m^−2^ yr^−1^ (Doskey & Ugoagwu, 1989; Lee *et al*., 1996; Cho *et al*., 2003; Lee & Booth, 2003), of which up to 3.5 mg P m^−2^ yr^−1^ could be in the form of InsP_6_ (Jackson & Linskens, 1982), whereas Hutchison & Barron (1997) and Read & Perez‐Moreno (2003) have highlighted the potential importance of pollen as a nutrient source for saprotrophic and mycorrhizal fungi, respectively. The present results raise the possibility that some epiphytic lichens could utilize InsP_6_ leached from pollen (Fig. 4) and thus might contribute to P cycling in forest canopies (cf. Read & Perez‐Moreno, 2003). Our initial demonstration of InsP_6_ release from lily pollen now requires confirmatory studies with pollen from ecologically relevant tree species together with analysis of canopy throughfall collected during the pollen season. Dwarf shrubs in the *Ericaceae* and *Empetraceae* that are characteristic of heathlands are predominantly insect‐pollinated and emit smaller quantities of pollen (Rodríguez‐Rajo *et al*., 2005), whereas pollen of the *Graminaceae* tested by Jackson *et al*. (1982) contained negligible InsP_6_. Hence, in the heathland habitats of *C. portentosa* pollen deposition might be a less significant source of nutrients for lichens.

Second, *Peltigera* spp contain cyanobacteria and have high N_2_‐fixation capacities, high P demands (Crittenden *et al*., 1994; McCune & Caldwell, 2009) and, according to predictions of Houlton *et al*. (2008), might be expected to have appreciable phosphatase activities. Furthermore, at the collection site *P. membranacea* grows amongst mosses on decaying tree stumps and boulders in deciduous woodland and so, like epiphytes, will also be exposed to throughfall but not stemflow.

The initial hydrolysis of InsP_6_ yielding InsP_5_ is probably catalysed by a specific phytase (Meek & Nicoletti, 1986; Shan *et al*., 1993). Our analytical results are consistent with Ins(1,2,4,5,6)P_5_ being the first hydrolysis product and with the lichen enzyme(s) responsible being a 3‐phytase, that is it first attacks the 3‐position on the inositol ring. 3‐phytases occur in other fungi (e.g. *Aspergillus ficuum*; Chen & Li, 2003) in contrast to 6‐phytases (yielding Ins(1,2,3,4,5)P5) and 5‐phytases (yielding Ins(1,2,3,4,6)P5) that occur in plants (Barrientos *et al*., 1994; Chen & Li, 2003; Jog *et al*., 2005), whereas all three enzyme types have been reported from bacteria (Sajidan *et al*., 2004; Puhl *et al*., 2008; Pontoppidan *et al*., 2012). Subsequent hydrolysis of lower‐order inositol phosphate esters could be at least partly attributable to nonspecific PMEs (Meek & Nicoletti, 1986); indeed, data presented by Kemme *et al*. (1999) suggest that dephosphorylation of InsP_3_ and InspP_2_ by *A. niger* phytase is much slower than that of InsP_6_–InsP_3_ and that final hydrolysis of InsP_1_ might be achieved exclusively by nonspecific PME enzymes (i.e. not by phytase). Peaks broadly consistent with InsP_2_–InsP_1_ increased in area with time indicating that dephosphorylation was ultimately complete, yielding inositol which could not be detected by the HPIC method. The proposed requirement for a specific phytase to initiate dephosphorylation of InsP_6_, but not that of InsP_5_–P_1_, is consistent with the observed progress in *E. prunastri* of InsP_6_ hydrolysis which remained linear, even when potentially competitive lower‐order inositol phosphates had accumulated in the assay medium (Fig. 2a–f). Nevertheless, *p*NPP, the analogous substrate for PME, did significantly inhibit phytase activity. This could result from either a direct interaction between *p*NPP and phytase or an inhibitory effect of Pi released from *p*NPP hydrolysis which was typically 50–100 times faster than InsP_6_ hydrolysis.

Phytase activity was pH dependent and hydrolysis products (e.g. InsP_5_ and InsP_4_) accumulated in the bathing medium. The hydrolysis of *p*NPP has similar properties which Bartlett & Lewis (1973), in their studies on mycorrhizal beech roots, considered indicative of extracellular PME activity. There is compelling evidence that PME activity in lichens is principally, if not entirely, associated with the fungus. Using an enzyme labelled fluorescent phosphatase substrate (ELF 97), Hogan *et al*. (2010a) showed that PME in *C. portentosa* was only detectable in the fungal symbiont and was concentrated in the outermost regions of the thallus. More recent work using ELF97 with other lichen species has shown PME activity to be restricted to the cortical cells (G. Brown & P. D. Crittenden, unpublished data). Furthermore, Hogan (2009) demonstrated PME activity in axenic cultures of *C. portentosa* and that it was positively related to the N : P mass ratio in the batch culture medium. It seems reasonable to suggest that extracellular phytase activity might have a distribution in the lichen thallus similar to that of surface‐bound PME. However, the two enzymes differed in the extent to which they could be washed from *E. prunastri* into the bathing medium. Approximately 20% of total lichen PME activity was attributable to enzyme(s) leached into the assay medium, whereas phytase activity in lichen washings alone was not significantly different from that in either non‐pretreated thalli plus washings or in thalli after 5 h washing (Fig. 3a); these results were confirmed in repeat experiments. It is possible that phytase has a substantially higher activity in free solution than in the thallus free‐space where it might be partially immobilized and/or in a different conformational state, such that in all lichen assays activity measured is largely that of the enzyme in free solution. This could make ecological sense if particulate matter such as pollen and microbial debris washed down from the canopy above is a principal source of InsP_6_ although, equally, investment of N in secreted proteins seems *prima facie* an unlikely strategy in a lichen‐forming fungus adapted to nutrient‐poor conditions.

There was evidence in *E. prunastri* that the rate of InsP_6_ consumption during short incubations (≤ 0.25 h) was atypically high. This putative artefact might have resulted from InsP_6_ precipitation or its adsorption onto cell wall surfaces. Note that using Beckett's (1995) estimates of the free‐space volume in lichens from xeric habitats (a mean of 32% of thallus volume), we estimate that the diluting effect of InsP_6_ diffusing into the thallus free‐space was probably negligible (0.3%). Pretreating thalli in InsP_6_ for 0.5 h before assays excluded this apparent artefact from rate measurements. It is unlikely that further nonenzymic loss of InsP_6_ could have been significant beyond 0.5 h for the following reasons: (1) InsP_6_ consumption proceeded at a constant rate thereafter; (2) response to temperature was linear between 5 and 30°C and the temperature quotient between 15 and 25°C (*Q*_10_ = 1.5) was broadly comparable with those for phytase activity in other microorganisms over the same temperature interval (e.g. *c*. 1.7 in *Erwinia carotovora* (Huang *et al*., 2009), *c*. 1.7 in *Janthinobacterium* sp. (Zhang *et al*., 2011), *c*. 1.4–2.5 in *Aspergillus* spp (Shivanna & Venkateswaran, 2014)); (3) some lichen samples had zero rates of activity (e.g. *C. portentosa*); and (4) activity in assays on *E. prunastri* thalli was similar to that in filtered thallus washings.

Rates of phytase activity in lichens are generally only a fraction of the rates of PME activity; similar proportionalities are evident in mycorrhizal roots and mycorrhizal fungi in axenic culture (Straker & Mitchell, 1986; Antibus *et al*., 1992, 1997; McElhinney & Mitchell, 1993; Colpaert *et al*., 1997). However, apparent *K*_m_ values for the two enzyme systems in lichens when calculated from whole thallus data are similar: the *K*_m_ value of 3.0 mM for phytase activity in *E. prunastri* can be compared with those for *p*NPP hydrolysis of 2.0 mM in the Antarctic species *Usnea sphacelata* (Crittenden *et al*., 2015), 2.2 mM in *C. portentosa* (Hogan *et al*., 2010a) and 8.9 mM in *Cladonia rangiferina* (Lane & Puckett, 1979). Further, the putative pH optima for phytase and PME activities in *E. prunastri* are similar (2.7 and 3.0, respectively; Lewis, 2012). Lichen species with high phytase activity also have high PME activity but the reverse is not necessarily true. Covariation between the two enzyme activities was striking when examined in a single species (*E. prunastri*) collected from locations with differing N deposition rates (Fig. 3b). Despite a growing body of evidence that upregulation of PME capacity in response to N enrichment is a general occurrence amongst a wide range of plants and microorganisms (Hogan *et al*., 2010b), neither phytase nor PME activities in *E. prunastri* were coherently related to wet N deposition (Fig. 3c). PME activity in lichens growing in treeless habitats is highly coupled to N deposition; PME activity in *C. portentosa* differed by a factor of 2 between British heathland sites with the lowest and highest N deposition rates (Hogan *et al*., 2010a) and in Antarctic fellfields PME activity in *Usnea sphacelata* differed by a factor of 6 between sites close to (2 km) and remote from (15 km) a penguin rookery (Crittenden *et al*., 2015). The weaker link between phosphatase activities and N deposition in *E. prunastri* might be explained by potential chemical modifications to rainfall that take place between interception by the tree canopy and deposition onto the surface of epiphytes.

The method used here for assaying phytase activity, which utilizes Chen & Li's (2003) HPIC‐based quantification of inositol phosphates, has distinct advantages over methods based on Pi release. It measures depletion of a specific naturally occurring substrate and the production of intermediate products of hydrolysis, thus confirming that substrate depletion is not an artefact. The method can be used with living material because the substrate and the measured hydrolysis products appear not to be taken up by the test samples. Although it is to some extent reassuring that rates of phytase activity in lichens measured by InsP_6_ depletion are similar to those documented for mycorrhizal fungi based on Pi release, it is perhaps surprising that rates in some lichen species are as high or higher than those in members of the Agaricomycotina, in which production of exoenzymes is a well‐understood strategy for exploitation of organic substrates. It is possible that measurements of phytase activity in fungal–root systems based on the rate of Pi release from InsP_6_ are underestimates due to the confounding effect of rapid active Pi uptake by physiologically active samples; a comparison of results produced by the two methods applied to the same test system is now needed to clarify this question. Disadvantages of the present method are that measurement of substrate depletion is generally less sensitive than measurement of product accumulation (cf. PME assay involving measurement of *p*NP production) and the analytical equipment is expensive to buy and maintain. Given the clear need for a robust phytase assay method, it is probable that the method used here will find application more widely in studies on plant root, fungal and soil systems.

## Supporting information

Please note: Wiley Blackwell are not responsible for the content or functionality of any supporting information supplied by the authors. Any queries (other than missing material) should be directed to the *New Phytologist* Central Office.

**Fig. S1 **Effect of substrate concentration on rate of InsP_6_ hydrolysis (Michaelis–Menten plot) in the apical 10 mm of *Evernia prunastri*.

**Table S1 **Replicate values of phytase and PME activities in *Evernia prunastri* and other selected lichens
