# Supplementary material for: Phytase activity in lichens
Source: New Phytol. 2015 May 12;208(2):544–54. doi: 10.1111/nph.13454 (PMC5029771; doi:10.1111/nph.13454)

***New Phytologist* Supporting Information**

Article title: **Phytase activity in lichens**
Authors: Niall F. Higgins and Peter D. Crittenden
Article acceptance date: 15 April 2015

The following Supporting Information is available for this article:

Fig. S1 Effect of substrate concentration on rate of InsP6 hydrolysis in the apical 10-mm of *Evernia prunastri*.

Table S1 Replicate values of phytase and PME activities in *Evernia prunastri* and other selected lichens (see separate Excel file)

Fig. S1 Effect of substrate concentration on rate of $InsP_6$ hydrolysis (Michaelis-Menten plot) in the apical 10-mm of *E. prunastri*. Rate of activity was determined from the difference between the quantities of $InsP_6$ hydrolysed in 0.5 and 5 h incubation periods. Assays were conducted in 1–10 mM $InsP_6$ at pH of 2.5 and 15°C in the dark. Plotted values are means (n = 6) ± 1 SEM. Effect of substrate concentration is significant at $P < 0.001$ (one-way ANOVA).

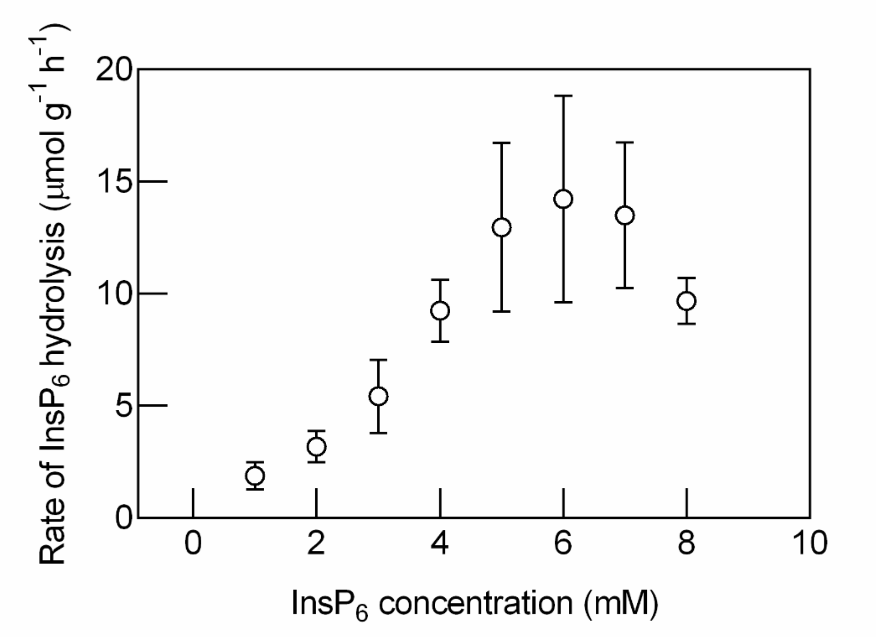

Supplement: Supplementary file 1 — Fig. S1 Effect of substrate concentration on rate of InsP 6 hydrolysis (Michaelis–Menten plot) in the apical 10 mm of Evernia prunastri. [file NPH-208-544-s001.pdf]
